# Trauma exposure and co‐occurring ICD‐11 post‐traumatic stress disorder and complex post‐traumatic stress disorder in adults with lived experience of psychiatric disorder

**DOI:** 10.1111/acps.13467

**Published:** 2022-07-13

**Authors:** Catrin Lewis, Katie Lewis, Alice Roberts, Bethan Edwards, Claudia Evison, Ann John, Alan Meudell, Patrick Parry, Holly Pearce, Natalie Richards, Ian Jones, Jonathan I. Bisson

**Affiliations:** ^1^ National Centre for Mental Health, Division of Psychological Medicine and Clinical Neurosciences Cardiff University School of Medicine Cardiff UK; ^2^ National Centre for Mental Health, PÂR Cardiff University School of Medicine Cardiff UK; ^3^ National Centre for Mental Health, Population Data Science Swansea University Medical School Swansea UK

**Keywords:** comorbidity, complex post‐traumatic stress disorder (CPTSD), post‐traumatic stress disorder (PTSD)

## Abstract

**Objective:**

To establish factors associated with ICD‐11 post‐traumatic stress disorder (PTSD) and complex PTSD (CPTSD) in a large sample of adults with lived experience of psychiatric disorder and examine the psychiatric burden associated with the two disorders.

**Methods:**

One thousand three hundred and five adults were recruited from the National Centre for Mental Health (NCMH) cohort. ICD‐11 PTSD/CPTSD were assessed with the International Trauma Questionnaire (ITQ). Binary logistic regression was used to determine factors associated with both PTSD and CPTSD. One‐way between‐groups analysis of variance was conducted to examine the burden associated with the two disorders in terms of symptoms of anxiety, depression, and psychological wellbeing. For post‐hoc pairwise comparisons, the Tukey HSD test was used, and the magnitude of between‐group differences assessed using Cohen's d.

**Results:**

Probable ICD‐11 CPTSD was more common than PTSD within the sample (PTSD 2.68%; CPTSD 12.72%). We found evidence that PTSD was associated with interpersonal trauma and household income under £20,000 a year. CPTSD was also associated with interpersonal trauma, higher rates of personality disorder, and lower rates of bipolar disorder. Those with probable‐CPTSD had higher levels of current anxiety and depressive symptoms and lower psychological wellbeing in comparison to those with probable‐PTSD and those with neither disorder.

**Conclusions:**

CPTSD was more prevalent than PTSD in our sample of people with lived experience of psychiatric disorder. Our findings indicate a need for routine screening for trauma histories and PTSD/CPTSD in clinical settings and a greater focus on the need for interventions to treat CPTSD.

**Significant outcomes**

- Complex post‐traumatic stress disorder (PTSD) was more prevalent than PTSD in our sample of people with lived experience of psychiatric disorder indicating an urgent need for interventions to treat CPTSD. Only a small proportion of those with probable CPTSD and PTSD reported having received the relevant diagnosis by a health professional (34% PTSD/22% CPTSD).
- We found evidence that PTSD was associated with interpersonal trauma and household income under £20,000 a year. CPTSD was associated with interpersonal trauma, higher rates of personality disorder, and lower rates of bipolar disorder.
- Those with probable‐CPTSD had higher levels of current anxiety and depressive symptoms and lower psychological wellbeing in comparison to those with probable‐PTSD and those with neither disorder.

**Limitations**

- Because of the wide‐ranging methods used to build the NCMH cohort and possible response biases associated with the follow‐up surveys, it cannot be assumed that the sample is representative of people with lived experience of psychiatric disorder.
- ICD‐11 PTSD/CPTSD was measured with use of a self‐report questionnaire, rather than a clinical interview. Although the ITQ has good psychometric properties, early work comparing the ITQ to the International Trauma Interview (ITI) suggests the two measure slightly different constructs.
- Despite the large sample, the number of participants with probable PTSD/CPTSD was relatively low, resulting in smaller sub‐samples for analysis, which may have impacted our findings.

## 1 INTRODUCTION

According to the 11th edition of the International Classification of Diseases (ICD‐11), post‐traumatic stress disorder (PTSD) is characterised by re‐experiencing a traumatic event in the here and now; avoidance of trauma reminders; and a sense of current threat.^1^ Approximately 60% of men and 50% of women experience at least one serious traumatic event through the course of their lives.^2^ The lifetime prevalence of trauma exposure is higher still among individuals with lived experience of psychiatric disorder.^3^, ^4^, ^5^, ^6^, ^7^, ^8^, ^9^ This may be indicative of an aetiological role of trauma in the development of disorders such as schizophrenia and bipolar disorder.^10^, ^11^ It is also a likely consequence of individuals with lived experience of psychiatric disorder having a greater risk of exposure to traumatic events such as interpersonal violence and victimisation.^12^

In a divergence from previous versions of the ICD and the Diagnostic and Statistical Manual 5th Edition (DSM‐5),^13^ ICD‐11 outlined two trauma‐related disorders, PTSD and complex PTSD (CPTSD). Both disorders are characterised by three core symptom clusters: (1) re‐experiencing the traumatic event; (2) deliberate avoidance of trauma reminders; and (3) persistent perceptions of heightened current threat. CPTSD is distinguished from PTSD based on symptoms that reflect the pervasive and enduring psychological changes that trauma exposure can precipitate. Termed ‘disturbances in self‐organisation’ (DSO), these symptoms fall into three clusters: (1) affective dysregulation; (2) negative self‐concept; and (3) disturbed relationships. According to the taxonomic structure of ICD‐11, it is not possible to be simultaneously diagnosed with both PTSD and CPTSD. The latter is most associated with repeated and prolonged trauma, but exposure‐type is a risk factor rather than a requirement for differential diagnosis.^14^ Exposure can occur in adulthood as well as childhood, but interpersonal trauma, that is trauma perpetrated by a person or people to harm others, during early development represents a particularly potent risk factor for CPTSD.^15^

In addition to evidence that childhood trauma predicts CPTSD, robust associations have been documented between traumatic exposures in childhood and other psychiatric disorders including schizophrenia, bipolar disorder, and personality disorder.^16^, ^17^ Despite this evidence, trauma‐history and traumatic stress symptoms often go undetected and untreated among individuals with primary psychiatric disorders other than PTSD.^18^ This has important clinical implications, given that undetected PTSD is associated with higher rates of mental and physical health problems; greater use of general medical and psychiatric services; higher levels of alcohol and substance misuse; and a poorer long‐term prognosis.^6^, ^9^, ^12^, ^19^, ^20^, ^21^ Previous studies have reported elevated rates of PTSD among individuals with primary diagnoses such as bipolar disorder and schizophrenia,^6^, ^9^, ^12^, ^19^, ^20^ but few studies have explored ICD‐11 PTSD/CPTSD among people with lived experience of psychiatric disorder.

The small number of studies that have examined ICD‐11 PTSD and/or CPTSD among participants with lived experience of psychiatric disorder have indicated that PTSD is prevalent, but CPTSD has received little attention. A study of 210 participants with bipolar disorder reported an ICD‐11 current PTSD prevalence of 31.8%, but CPTSD was not measured.^22^ Looking at patterns of *ICD‐11* PTSD comorbidity among a nationally representative sample of 530 older adults in the United States, nearly 80% of the sample with PTSD reported at least one comorbid disorder, with the highest level of comorbidity with major depressive disorder (MDD) and alcohol use disorder (AUD).^23^ Again, this study did not look at CPTSD. In a small sample of 165 Danish psychiatric outpatients, 94% of participants reported having experienced at least one traumatic event, CPTSD was more common than PTSD (PTSD 8%; CPTSD 36%) and had considerable overlap with ICD‐10 affective, anxiety, personality, adjustment, and behavioural and emotional disorders.^24^ Finally, a study of sentenced male prisoners in the United Kingdom (*N* = 221) found that CPTSD was significantly associated with diagnoses of depression, substance misuse, and psychosis, following adjustment for potential confounders.

It is evident that the studies of ICD‐11 PTSD/CPTSD to date have been based on relatively small samples. In psychiatric research, a variety of methods have been used to determine participant diagnoses. The most robust methods rely on full clinical assessment or validated research interviews administered by trained personnel. Although these methods are the gold‐standard, they are resource intensive and demanding of participant time, which inevitably impacts sample sizes. Despite limitations to self‐reports of psychiatric diagnoses, such as participants not being aware of having a diagnosis, or issues of misinterpretation or inaccurate recall, recent studies have indicated that self‐endorsement of diagnoses gives a good indicator of the presence of psychiatric disorder, thereby facilitating epidemiological research on a large scale.^25^, ^26^, ^27^

The elevated risk of trauma exposure, combined with shared vulnerability factors for the development of PTSD and other psychiatric disorders results in an increased risk of PTSD for those with other primary mental health diagnoses.^12^, ^20^ Despite these findings, PTSD often goes undetected and untreated in this population because of symptom overlap and a tendency for clinicians not to fully examine traumatic stress symptoms during clinical assessment.^3^ Since comorbid PTSD and CPTSD are common and understudied among people with lived experience of psychiatric disorder, it is necessary to determine the extent of co‐occurrence across different diagnostic and demographic groups to establish indicators that may help identify those at greatest risk, where asking about trauma histories and traumatic stress symptoms might be particularly helpful. There is a need for research to better understand ICD‐11 PTSD/CPTSD among people with lived experience of psychiatric disorder to improve differential diagnosis, facilitate detection of comorbid disorder, and inform treatment planning.

### 1.1 Aims of the study

The aims of this study were to establish factors associated with both PTSD and CPTSD in a large sample of adults with self‐reported lived experience of psychiatric disorder. Based on previous literature, we hypothesised that both PTSD and CPTSD would be associated with younger age,^28^, ^29^ female gender,^28^, ^29^ and current income.^30^ Also based on findings to date, we hypothesised that CPTSD would be associated with interpersonal trauma (i.e., those perpetrated by a person or people to harm another person or people)^31^ and trauma in childhood.^32^, ^33^, ^34^ Based on reported associations between traumatic exposures in childhood and schizophrenia/other psychotic disorders, bipolar disorder, and personality disorder,^16^, ^17^ we hypothesised these would be associated with CPTSD. In line with previous studies, we hypothesised that participants with CPTSD would report a greater burden of current symptoms of anxiety and depression, and poorer psychological wellbeing compared with those with PTSD or no diagnosis.^35^

## 2 METHOD

### 2.1 Data source

Data were obtained from the National Centre for Mental Health (NCMH), a Welsh Government‐funded Research Centre that investigates psychiatric disorders across the lifespan. The Centre is operated by Cardiff, Swansea, and Bangor Universities, in partnership with the National Health Service (NHS) across Wales and England. NCMH hosts a cohort of participant volunteers that includes individuals with lived experience of a psychiatric disorder. Participants were recruited into the cohort on a rolling basis from 2011 using a variety of systematic approaches in primary and secondary health care services, including (1) the identification of potential participants by clinical care teams; and (2) screening of clinical notes. Non‐systematic recruitment approaches have included advertising via media and social media and engaging third‐sector organisations to promote the research. Participants joined the cohort by taking part in a face‐to‐face interview with a researcher or completing an online assessment. Written informed consent was obtained from all participants. On joining the cohort, participants provided clinical information (including mental health diagnoses) and demographic data (e.g., gender, age, ethnicity, employment). Psychiatric diagnoses were determined by asking participants to self‐report diagnoses received by a health professional or for which they had received treatment. All study procedures were given a favourable ethical opinion by Wales Research Ethics Committee 2.

### 2.2 Sample

**FIGURE 1:**
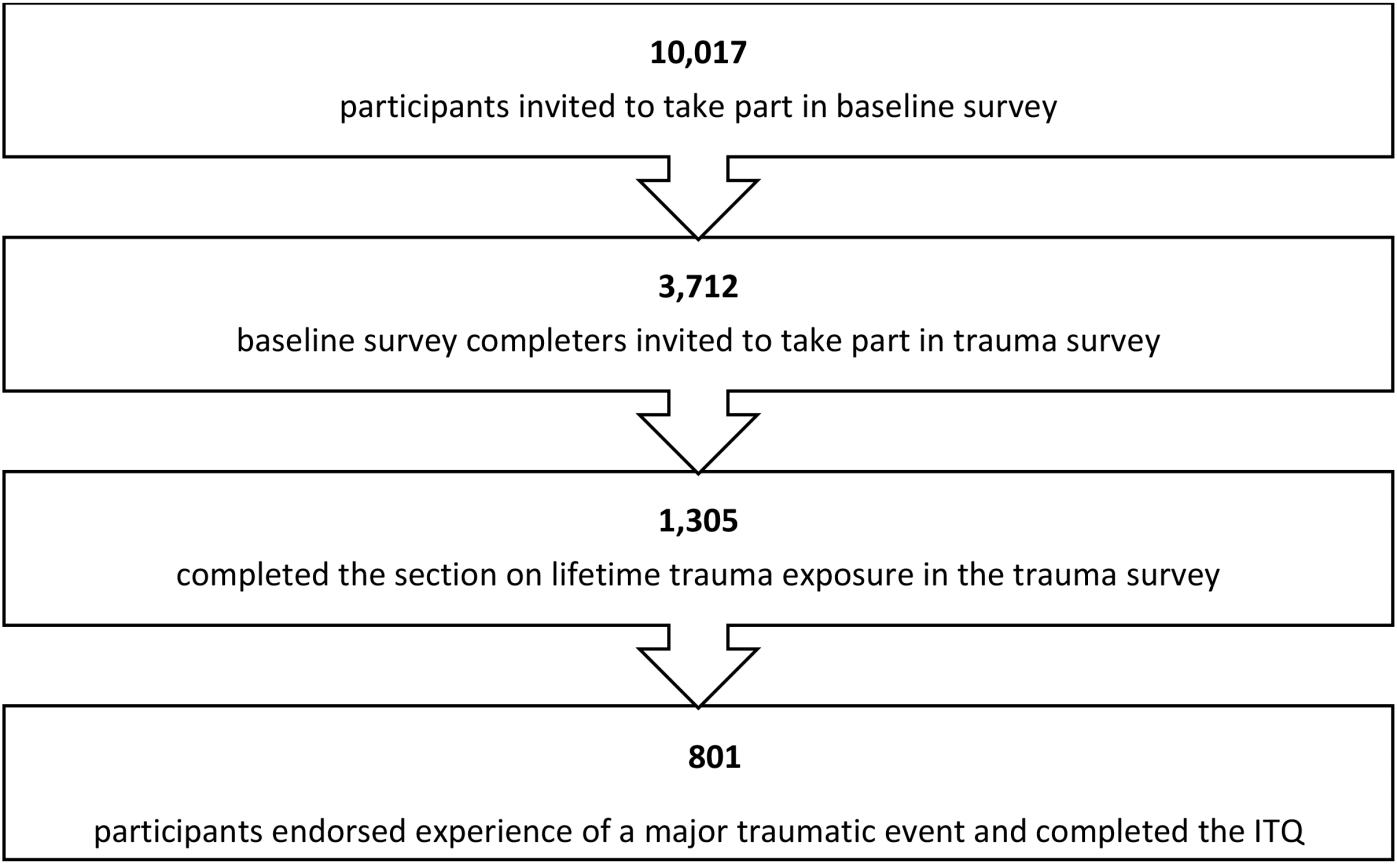
Participant flow. ITQ, International Trauma Questionnaire

In June 2020, all members of the NCMH cohort aged 18 years or older with lived experience of a psychiatric disorder, who had consented to be contacted for future research and provided an email address (*n* = 10,017) were invited to complete an online survey that assessed the impact of the COVID‐19 pandemic on mental health. This baseline COVID‐19 survey included questions on demographic variables (gender, age, employment, income, living arrangements), mental health and psychiatric service use, and questions related to the COVID‐19 pandemic. In November 2020, a follow‐up survey was sent to the 3712 participants who had completed the baseline survey (see Figure 1 for participant flow). The follow up survey repeated questions from the baseline survey and asked additional questions specifically related to traumatic stress. The sample for analysis consisted of all participants who completed questions on lifetime trauma exposure and PTSD as part of this survey. COVID‐19 related PTSD measured separately and findings are reported elsewhere.^36^

### 2.3 Data collection and outcome measures

#### 2.3.1 Demographic and clinical information

Self‐reported age, gender, ethnicity, household income and employment were captured in the baseline COVID‐19 survey. Changes in finances and employment were determined in the follow‐up survey. Mental health diagnoses were collected as part of the baseline survey with the question ‘what mental health or neurodevelopmental diagnosis or condition have you been given or received treatment for?’ with instructions to select from a list of options (see Dataset S1) or endorse the option of ‘other’ and provide a free‐text response. Self‐reported lifetime diagnoses were grouped into the following composite categories: anxiety disorder; autism spectrum disorder; bipolar disorder; depressive disorder; personality disorder; PTSD; and CPTSD.

#### 2.3.2 Trauma exposure and PTSD

Participants were asked whether they had ever experienced a ‘major’ traumatic event and, if so, were asked to provide additional information. To take a stringent approach to the assessment of PTSD and to anchor reported symptoms to a single event as required in the assessment of ICD‐11 PTSD, traumatic exposure was determined by asking participants to provide a free text response to the following: ‘please identify the experience that troubles you most and answer the questions in relation to this experience’. The descriptions of traumatic events were coded against a list of traumas compiled from the Life Events Checklist for DSM‐5 (LEC)^37^ and the International Trauma Exposure Measure (ITEM),^38^ with the addition of traumatic events reported by the cohort that were not covered by the LEC (e.g., suicide attempts). This resulted in a comprehensive list of traumatic events. When participants reported multiple events, the first listed trauma was taken as the ‘most troubling’ event. Two researchers independently coded each of the descriptions. If coders disagreed on any of the classifications, this was discussed with a third researcher and a consensus was reached.

All participants who endorsed a traumatic event were invited to complete the International Trauma Questionnaire (ITQ)^39^ in relation to their most troubling experience. The ITQ asks participants to indicate how much they have been bothered by six core PTSD symptoms in the past month using a five‐point Likert scale ranging from 0 (‘Not at all’) to 4 (‘Extremely’). Two symptoms reflect the re‐experiencing cluster of symptoms (upsetting dreams and flashbacks); two the avoidance cluster (avoiding internal or external reminders); and two the sense of current threat cluster (being hypervigilant or easily startled). A set of six questions capture DSO symptoms to enable differential detection of probable CPTSD. Two items reflect each of the three clusters: (1) ‘affective dysregulation’; (2) ‘negative self‐concept’; and (3) ‘disturbed relationships’. Additional items capture functional impairment associated with both PTSD and DSO symptoms in three domains: (1) relationships and social life; (2) work or ability to work; and (3) other important aspects of life. Cronbach's alpha (*α*) for the current study was 0.94. Participants were considered to fulfil the criteria for probable ICD‐11 PTSD if a qualifying trauma was reported, a score of ≥2 (‘Moderately’) was obtained for at least one of two symptoms from each of the three symptom clusters, and at least one of the functional impairment items was endorsed. Participants were considered to fulfil the criteria for probable ICD‐11 CPTSD if they satisfied the criteria for PTSD, in addition to scoring ≥2 (‘Moderately’) for at least one symptom from each of the three DSO symptom clusters.

#### 2.3.3 Symptoms of current depression

Symptoms of current depression were measured by the nine‐item Patient Health Questionnaire‐9 (PHQ‐9).^40^ Respondents indicated the frequency of symptoms in the previous 2 weeks on a four‐point Likert‐scale, ranging from 0 (not at all) to 3 (nearly every day). Total scores can range from 0 to 27. *α* for the current study was 0.92.

#### 2.3.4 Symptoms of current anxiety

Symptoms of current anxiety were measured by the seven‐item Generalised Anxiety Disorder Assessment‐7 (GAD‐7).^41^ Respondents indicated the frequency of anxiety symptoms in the previous 2 weeks on a four‐point Likert‐scale, ranging from 0 (not at all) to 3 (nearly every day). Total scores can range from 0 to 21. *α* for the current study was 0.92.

#### 2.3.5 Psychological wellbeing

Psychological well‐being was measured by the WHO‐5 Well‐being Index (WHO‐5).^42^ Respondents rated five statements on a Likert‐scale, ranging from 5 (‘all of the time’) to 0 (‘at no time’). Total scores can range from 0 to 25 with higher scores indicating greater well‐being. *α* for the current study was 0.90.

### 2.4 Statistical procedures

All analyses were conducted using Stata version 16.^43^ Firstly, the point prevalence of probable ICD‐11 PTSD and CPTSD (according to the ITQ) was ascertained within the sample. To address the first aim of the study, binary logistic regression was used to examine the association between key demographic and trauma related variables and having probable (1) CPTSD and (2) PTSD in two separate models. Based on previous literature and decided a priori, variables of interest were age; gender; current income; interpersonal trauma (i.e., those perpetrated by a person or people to harm another person or people); trauma in childhood; and self‐reported lifetime diagnoses of anxiety disorder; autism spectrum disorder; bipolar disorder, depressive disorder; personality disorder; and schizophrenia/other psychotic disorders. The Holm method was used to adjust p values to account for multiple testing.^44^ To address the second aim and examine whether mean levels of current depression, anxiety, and psychological wellbeing differed across the three groups (probable CPTSD; probable PTSD; and no CPTSD/PTSD), one‐way between‐groups analysis of variance (ANOVA) was conducted. For post‐hoc pairwise comparisons, the Tukey HSD test was used, and the magnitude of between‐group differences assessed using Cohen's d with values of 0.4 or less indicating a small effect; 0.5–0.7 a medium effect and 0.8 or greater a large effect.^45^

## 3 RESULTS

The sample for analysis were 1305 participants (41.6% of those invited) who completed the section on lifetime trauma exposure in the survey. Using variables collected at the point of entry into the NCMH cohort with complete or near‐complete data, we found that non‐response to the trauma survey was associated with younger age, being male, never having been employed, minority ethnicity, diagnosis of bipolar disorder and diagnosis of schizophrenia. We did not find evidence of an association between diagnosis of PTSD and/or CPTSD and non‐response (see Dataset S1). Most of the current sample (70.7%) completed the survey between 5th November and the 11th November 2020, with the rest of the sample completing the survey between 12 November 2020 and 2 January 2021.

### 3.1 Sample characteristics

The mean age of participants was 46.7 (SD = 15.5) and 75.3% (*n* = 983) were female. Most participants were white (95.4%); 51.1% had a household income over £20,000, and all participants self‐reported lived experience of at least one psychiatric disorder. Sample characteristics are presented in Table 1.

**TABLE 1:** Sample characteristics

	Total	CPTSD	PTSD	No PTSD/PTSD
	(*n* = 1305)	(*n* = 166)	(*n* = 35)	
				(*n* = 1104)
Variable	*N* (%S) or mean (SD)	*N* (%) or mean (SD)	*N* (%) or mean (SD)	*N* (%) or mean (SD)
Age	46.71 (15.52)	43.19 (14.56)	46.06 (15.03)	47.26 (15.63)
Gender				
Female	983 (75.33)	134 (80.72)	27 (81.82)	822 (74.86)
Male	297 (22.78)	26 (15.66)	6 (18.18)	265 (24.13)
Other/missing	25 (1.92)	6 (0.04)	2 (5.71)	17 (1.54)
Ethnicity				
White	1245 (95.40)	160 (96.39)	33 (94.29)	1052 (95.29)
Ethnic minority	43 (3.30)	5 (3.01)	1 (2.86)	37 (3.35)
Missing	17 (1.30)	1 (0.60)	1 (2.86)	15 (1.36)
Household income				
Up to £20,000	493 (37.78)	89 (51.81)	22 (66.67)	382 (34.79)
£20,000+	667 (51.11)	63 (37.95)	10 (30.30)	594 (54.10)
Missing	145 (1.11)	14 (8.43)	3 (8.57)	128 (11.59)
Self‐reported mental health diagnoses				
Bipolar disorder	142 (10.88)	10 (6.02)	5 (15.15)	127 (11.57)
Psychotic disorder	99 (7.59)	12 (7.23)	2 (6.06)	85 (7.74)
Depressive disorder	1006 (77.09)	129 (77.71)	26 (78.79)	851 (77.50)
Anxiety disorder	821 (62.91)	118 (71.08)	20 (60.61)	683 (62.20)
Personality disorder	156 (11.95)	48 (28.92)	5 (15.15)	103 (9.38)
PTSD	194 (14.87)	73 (43.98)	12 (36.36)	109 (9.93)
Complex PTSD	66 (5.06)	36 (21.69)	6 (18.18)	24 (2.19)
Autism spectrum disorder	81 (6.21)	17 (10.24)	3 (9.09)	61 (5.56)
*Worst traumatic event*				
Directly experiencing assaultive violence				
Physical assault	22 (1.69)	7 (4.22)	0 (0)	15 (1.37)
Sexual assault	143 (10.96)	53 (31.93)	12 (36.36)	78 (7.10)
Child abuse	71 (5.44)	30 (18.07)	8 (24.24)	33 (3.01)
Domestic abuse	50 (3.83)	16 (9.64)	5 (15.15)	29 (2.64)
Directly experiencing traumatic events				
Disaster/terrorism	6 (0.46)	0 (0)	2 (6.06)	4 (0.36)
Combat exposure	16 (1.22)	9 (5.42)	2 (6.06)	5 (0.46)
Transportation accident	71 (5.44)	30 (18.07)	8 (24.24)	33 (3.01)
Traumatic childbirth	14 (1.07)	3 (1.81)	1 (3.03)	10 (0.91)
Causing death/injury to another	1 (0.08)	1 (0.60)	0 (0)	0 (0)
Life threatening illness or injury	34 (2.61)	3 (1.81)	1 (3.03)	30 (2.73)
Other serious accident	4 (0.31)	0 (0)	2 (6.06)	2 (0.18)
Other life‐threatening trauma	4 (0.31)	1 (0.60)	0 (0)	3 (0.27)
Suicide attempt	11 (0.84)	2 (1.20)	2 (6.06)	7 (0.64)
Experience of mental illness	77 (5.90)	2 (1.20)	0 (0)	75 (6.83)
Witnessing traumatic events				
Unexpected death	7 (0.54)	1 (0.60)	0 (0)	6 (0.55)
Death of someone close	7 (0.54)	1 (0.60)	0 (0)	6 (0.55)
Finding or seeing a dead body	5 (0.38)	1 (0.60)	0 (0)	4 (0.36)
Life threatening trauma	20 (1.53)	5 (3.01)	0 (0)	15 (1.37)
Violence/attack	14 (1.07)	6 (3.61)	0 (0)	8 (0.73)
Learned about traumatic events				
Physical assault				
Sexual assault	1 (0.08)	0 (0)	0 (0)	1 (0.09)
Accident	3 (0.23)	0 (0)	0 (0)	3 (0.27)
Violent/accidental death	27 (2.07)	4 (2.41)	2 (6.06)	21 (1.91)
Life threatening illness/injury	16 (1.23)	4 (2.41)	0 (0)	12 (1.09)
Other non‐life threatening	1 (0.08)	0 (0)	0 (0)	1 (0.09)
Bereavement				
Death of child or grandchild	7 (0.54)	2 (1.20)	0 (0)	5 (0.46)
Pregnancy loss/neonatal death	10 (0.77)	2 (1.20)	0 (0)	8 (0.73)
Death of partner (not witnessed)	10 (0.77)	1 (0.60)	0 (0)	9 (0.82)
Death of parent	33 (2.53)	3 (1.81)	0 (0)	30 (2.73)
Other death	22 (1.69)	0 (0)	0 (0)	22 (2.00)
Divorce/family issues	22 (1.69)	0 (0)	0 (0)	22 (2.00)

Abbreviations: CPTSD, complex PTSD; PTSD, post‐traumatic stress disorder.

### 3.2 Trauma exposure and ICD‐11 PTSD/CPTSD

In total, 61.4% (*n* = 801) of participants reported having experienced a ‘major’ traumatic event at some point in their lives. The most prevalent traumatic exposure was sexual assault (11.0%; *n* = 143). A full list of the self‐reported traumatic exposures is presented in Table 1. 39.4% (*n* = 514) of the sample self‐reported a traumatic event that was judged to be PTSD qualifying according to ICD‐11 criteria as judged by two researchers. 2.7% (*n* = 35) met criteria for probable ICD‐11 PTSD and 12.7% (*n* = 166) for probable ICD‐11 CPTSD. Only 22% of those with probable CPTSD reported having received the relevant diagnosis from a health professional and 34% of those with PTSD.

### 3.3 Factors associated with ICD‐11 PTSD/CPTSD

Results of multivariate binary logistic regression analyses are presented in Table 2. We found evidence that PTSD was associated with interpersonal trauma and lower household income. CPTSD was also associated with interpersonal trauma, and unlike PTSD, higher rates of personality disorder, and lower rates bipolar disorder. We did not find evidence of either PTSD or CPTSD being associated with gender; age; a worst traumatic event that happened in childhood (rather than adulthood); diagnosis of autism spectrum disorder; depressive disorder; or psychotic disorder.

**TABLE 2:** Results of multivariate binary logistic regressions examining variables associated with PTSD (Model 1) or CPTSD (Model 2)

	Model 1		Model 2			
	PTSD versus no PTSD/CPTSD		CPTSD versus no PTSD/CPTSD			
Variable	OR	95% CI	*p*	OR	95% CI	*p*
Age	0.10	0.96–1.02	0.730	1.00	0.99–1.01	0.979
Gender	1.48	0.46–4.72	0.512	1.12	0.65–1.92	0.692
Income	3.07	1.30–7.26	0.011	1.52	1.02–2.29	0.042
Childhood trauma	1.15	0.48–2.75	0.756	1.19	0.75–1.90	0.461
Interpersonal trauma	4.66	1.77–12.26	0.002	8.25	5.15–13.20	<0.001
Anxiety disorder	0.63	0.27–1.48	0.295	1.29	0.82–2.01	0.271
Autism spectrum disorder	1.18	0.25–5.66	0.835	1.25	0.58–2.66	0.571
Bipolar disorder	1.60	0.48–5.31	0.443	0.28	0.15–0.68	0.005
Depressive disorder	0.75	0.29–1.98	0.563	0.65	0.39–1.08	0.093
Personality disorder	0.68	0.24–1.95	0.472	2.76	1.68–4.53	<0.001
Psychotic disorder	0.55	0.12–2.51	0.443	0.84	0.39–1.81	0.654

*Note*: Age—continuous; gender coded as 0 = male, 1 = female; income coded as 1 = gross family income under £20,000 a year, 0 = household income £20,000 or more; childhood trauma 1 = yes, 0 = no; interpersonal trauma 1 = yes, 0 = no; lived experience of anxiety disorder coded as 1 = yes, 0 = no; lived experience of autism spectrum disorder coded as 1 = yes, 0 = no; lived experience of bipolar disorder coded as 1 = yes, 0 = no; lived experience of depressive disorder coded as 1 = yes, 0 = no; lived experience of personality disorder coded as 1 = yes, 0 = no; lived experience of psychotic disorder coded as 1 = yes, 0 = no.

Abbreviations: CPTSD, complex PTSD; PTSD, post‐traumatic stress disorder.

### 3.4 Symptoms of depression, anxiety, and psychological wellbeing

Results of the one‐way between groups ANOVAs, conducted to examine whether mean levels of depression, anxiety, and psychological wellbeing differed across the three groups (probable CPTSD; probable PTSD; and no CPTSD/PTSD), are presented in Table 3. Within the sample, mean (SD) scores on the PHQ‐9 were 20.03 (5.35) in the CPTSD group; 12.66 (5.42) in the PTSD group and 11.13 (7.29) in the no PTSD/CPTSD group. On the GAD‐7, mean (SD) scores were 15.60 (4.49) for the CPTSD group; 11.77 (5.26) for the PTSD group; and 9.09 (6.13) for those with no PTSD/CPTSD. Mean (SD) scores on the WHO‐5 were 4.19 (3.33) in the CPTSD group; 7.27 (5.13) in the PTSD group; and 8.91 (5.40) in the no PTSD/CPTSD group. We found statistically significant differences between the three groups in terms of mean PHQ‐9, GAD‐7, and WHO‐5 scores (*p*s <0.001). Post‐hoc comparisons using the Tukey HSD test indicated those with probable CPTSD had higher levels of current depression symptoms (*d* = 1.26) and current anxiety symptoms (*d* = 1.10) and lower levels of psychological wellbeing (*d* = 0.91) compared with the no PTSD/CPTSD group. We also found that those with probable CPTSD had higher levels of current depression symptoms (*d* = 1.37) and current anxiety symptoms (*d* = 0.83) and lower levels of psychological wellbeing (*d* = 0.84) compared with the probable PTSD group. These were large effects.^45^

**TABLE 3:** One way between groups ANOVA conducted to examine whether mean levels of depression, anxiety, and psychological wellbeing differed across the three groups (probable CPTSD; probable PTSD; and no CPTSD/PTSD)

	Symptoms of current depression (PHQ‐9)				Symptoms of current anxiety (GAD‐7)				Psychological wellbeing (WHO‐5)			
Diagnostic group according to assessment with ITQ	Mean	SD	*F*	*p*	Mean	SD	*F*	*p*	Mean	SD	*F*	*p*
Probable PTSD (*n* = 35)	12.66	5.42	107.58	<0.001	11.77	5.26	77.36	<0.001	7.27	5.13	56.59	<0.001
Probable CPTSD (*n* = 166)	20.03	5.35			15.60	4.49			4.19	3.33		
No PTSD/CPTSD (*n* = 1104)	11.13	7.29			9.09	6.13			8.91	5.40		

Abbreviations: ANOVA, analysis of variance; GAD‐7, generalised anxiety disorder 7; ITQ, International Trauma Questionnaire; PHQ‐9, Patient Health Questionnaire 9; WHO‐5, WHO‐5 Well‐being Index.

## 4 DISCUSSION

This study aimed to examine factors associated with PTSD and CPTSD in a large sample of adults with lived experience of psychiatric disorder and to ascertain the burden associated with the two disorders in terms of current symptoms of anxiety, depression, and psychological wellbeing. To address these aims, we first determined the point prevalence of probable ICD‐11 PTSD and CPTSD within the sample, according to the ITQ. We found a greater prevalence of probable CPTSD than PTSD (2.68% PTSD; 12.72% CPTSD), which contradicts findings from a nationally representative general population sample in Israel which found a higher prevalence of PTSD (9.0% PTSD; 2.6% CPTSD),^46^ and a population‐based sample in the United States (3.4% PTSD; 3.8% CPTSD)^35^ which found a broadly similar prevalence of the two disorders. However, it is consistent with findings from UK samples of trauma exposed participants (5.3% PTSD; 12.9% CPTSD)^31^ and treatment seeking Veterans (14% PTSD; 56.7% CPTSD),^47^ and a Danish sample of psychiatric outpatients (PTSD 8%; CPTSD 36%).^24^ Although some early work assumed that CPTSD was a subtype of PTSD,^48^ work such as our own that found evidence of CPTSD being the predominant disorder, contradicts this view. Given the nature of our sample and a dominance of female participants, it is perhaps surprising that the prevalence of CPTSD was not greater. However, DSO symptoms such as difficulties with emotion regulation, affective instability, and disturbed self‐concept, overlap with characteristic symptoms of many of the psychiatric disorders represented in the cohort, and may previously have been targeted by treatment in the context of these co‐occurring conditions. However, it is worth noting that our sample was not a representative sample of people with lived experience of psychiatric disorder given the voluntary nature of participation.

In terms of factors associated with ICD‐11 PTSD and CPTSD, we found evidence that interpersonal trauma (e.g., sexual and physical assault) was associated with both disorders. This contrasts with findings from a study of trauma exposed participants in the United Kingdom, which found that interpersonal trauma was uniquely associated with CPTSD.^31^ We found evidence that lower income (under £20,000 a year) was associated with PTSD but not with CPTSD. This adds to mixed findings related to income and PTSD/CPTSD in the literature.^32^, ^49^, ^50^ However, it is worth noting that we captured household income versus personal income. We did not find evidence of associations between PTSD or CPTSD and sex or age. This contrasts with findings from studies that found an association between both disorders and being younger and female^28^, ^29^ but these findings have not always been replicated.^31^ We did not find evidence of an association between PTSD or CPTSD and self‐reported childhood trauma as the most troubling traumatic exposure. This contrasts with many previous studies, with an association between childhood trauma and CPTSD being particularly well replicated.^32^, ^33^, ^34^ This may be because of our focus on the ‘most troubling’ traumatic event. Those with a ‘most troubling’ event that occurred in adulthood may also have had a history of childhood trauma that we did not consider. Secondly, our sample was of people with lived experience of psychiatric disorder and rates of childhood trauma were generally high. It may be that a history of childhood trauma differentiates between those with and without CPTSD in the general population to a greater extent than it does among people with lived experience of psychiatric disorder.

We found evidence that CPTSD (but not PTSD) was associated with lived experience of personality disorder. This may be because of an aetiological contribution of trauma, especially prolonged childhood trauma, to the development of both disorders.^51^, ^52^, ^53^ Another possible explanation is that DSO symptoms are inherently cross‐diagnostic especially when assessed by a self‐report measure, and controversy has surrounded the overlap between ICD‐11 DSO symptoms and borderline personality disorder (BPD). Another possible explanation is participants having been diagnosed with BPD rather than CPTSD before diagnosis of the latter existed. Like CPTSD, diagnostic features of BPD include disturbed interpersonal relationships, problems with emotion regulation, and a disturbed sense of self. Although in BPD self‐concept is unstable, relationships are volatile and sense of self fluctuates, whereas in CPTSD they are stable, relationships are avoided, and sense of self is negative.^13^ CPTSD was associated with lower rates of bipolar disorder. This finding should be interpreted with consideration of the fact that everyone in the sample had lived experience of psychiatric disorder. However, there is no clear explanation for this finding, and it would be interesting to determine whether it can be replicated in other samples.

We found evidence that probable CPTSD was associated with higher levels of current depression and anxiety symptoms, and lower psychological wellbeing in comparison to those with probable PTSD and no PTSD/CPTSD. This is consistent with the findings of a previous study in a nationally representative sample of the US population, which used identical measures to our own study.^35^ It is also consistent with a study of trauma exposed participants in the United Kingdom, which found that current major depressive disorder and GAD were more strongly associated with CPTSD than PTSD.^31^ This adds weight to the evidence that CPTSD is associated with greater psychiatric burden than PTSD, as indicated by previous work.^42^ It also furthers our understanding of CPTSD as a more impairing comorbidity than PTSD.

### 4.1 Strengths and limitations

To our knowledge, this is the largest study to date to explore factors associated with ICD‐11 PTSD and CPTSD within a cohort of individuals with lived experience of psychiatric disorder. We included a broad spectrum of participants with a range of psychiatric diagnoses, varying in terms of complexity and severity. We anchored the screening tool to specific traumatic events as required for the valid assessment of ICD‐11 PTSD and CPTSD. Reported traumas were categorised and judged by two researchers independently to determine whether they met ICD‐11 criteria for a PTSD qualifying event, which increased confidence in the validity of our results.

However, the findings should be considered in the context of study limitations. Firstly, data was collected as part of a survey conducted during the COVID‐19 pandemic, which may have impacted our results. That said, we asked about COVID‐19 related trauma separately^36^ and requested participants focus on traumatic events other than COVID‐19 in this section of the survey. ICD‐11 PTSD/CPTSD was measured with use of a self‐report questionnaire, rather than a clinical interview. Although the ITQ has good psychometric properties, early work comparing the ITQ to the International Trauma Interview (ITI) suggests the two measure slightly different constructs.^54^ Despite the large sample, the small number of participants with PTSD/CPTSD resulted in smaller sub‐samples for analysis, which may have impacted our findings. Participants were asked to self‐report psychiatric diagnoses received from a health professional and it is possible the true frequency was over or under reported. In addition, we did not have data related to the duration of psychiatric disorder or care received. The restrictions posed by the COVID‐19 pandemic dictated that the research was conducted exclusively online. Only members of the NCMH cohort with an email address were invited to take part. This may have sub‐optimally represented older participants and participants from very low‐income households who are less likely to be digitally active.^55^ There were additional reasons to suspect the sample was not truly representative of people with lived experience of psychiatric disorder. This included the wide‐ranging recruitment strategies used to build the cohort from which participants were drawn, over‐representation of female participants, and evidence of response bias in terms of those who completed the survey. This resulted in a sample that is unlikely to be fully generalisable. Although it is a strength that the sample included participants with diagnoses such as personality disorder and schizophrenia, who are traditionally under‐represented in research,^56^ the findings should not be over‐interpreted. It is also worth noting the sample was 95.4% White, which also limits generalisability. However, a high proportion of participants were recruited in Wales, which has a low level of ethnic diversity and an estimated 92.2% of the population identify as White Welsh/British.^57^ Another limitation was that we only measured current traumatic stress symptoms and the rates of undetected past‐PTSD/CPTSD were therefore unknown. Since CPTSD is often more chronic than PTSD, measuring lifetime history rather than current symptoms may have yielded different findings. In addition, participants were asked whether they experienced what they perceived to be a ‘major’ traumatic event and were only required to elaborate if a positive response was given. This may have resulted in under‐reporting of events that met criteria for PTSD on the basis that participants did not perceive the event to be ‘majorly traumatic’. In addition, we did not explore the impact of multiple traumas and the extent of symptoms may have been under‐reported. Finally, the research was cross‐sectional, and all findings represent associations.

## 5 RESEARCH IMPLICATIONS

Future research using longitudinal designs is required to disentangle cause and effect, aid determination of factors underlying comorbidity, and explore the long‐term implications of PTSD and CPTSD as comorbid disorders. Studies using diagnostic interviews to detect PTSD and CPTSD and more robust methods of ascertaining trauma histories would be desirable. It would also be interesting to determine whether the extent of comorbidity in terms of the number of psychiatric diagnoses impacts an association with PTSD/CPTSD. This work points to a need for the development, evaluation, and dissemination of appropriate treatments for comorbid PTSD/CPTSD. Although there is a large evidence‐base related to the treatment of PTSD,^58^ there is far less research on the treatment of comorbid and/or complex PTSD and this merits further attention. In the case of CPTSD, work is urgently needed to develop explanatory models for the onset and maintenance of the disorder and for these to inform novel interventions that can be evaluated. Given mounting evidence that CPTSD is more common than PTSD, especially among people with lived experience of other psychiatric disorders, there is a need for interventions that are scalable, accessible, and easy to deliver. Since there is a growing body of evidence to support the efficacy of digital guided self‐help interventions for PTSD,^59^ there may be value in the development and evaluation of similar interventions for CPTSD.

## 6 CLINICAL IMPLICATIONS

Our findings indicate the potential value of asking about trauma histories and traumatic stress symptoms in psychiatric settings. This is of particular importance given our findings that PTSD and CPTSD are associated with a higher burden of symptoms compared with those without these disorders, and indicators from previous research that those with comorbid PTSD/CPTSD are likely to exhibit greater impairment, poorer prognosis, and increased use of general medical and mental health services.^6^, ^9^, ^12^, ^19^, ^20^, ^21^, ^23^, ^35^ Given the evidence that CPTSD is the predominant disorder within clinical samples, there is a particular need to look out for DSO symptoms. The ITQ is a validated self‐report measure that can be used to identify probable ICD‐11 PTSD/CPTSD and determine those in need of intervention.^39^ In our sample, only a small proportion of those with probable CPTSD and PTSD reported having received the relevant diagnosis by a health professional (34% PTSD/22% CPTSD). In comparison, 82% of those who scored above a cut off taken to indicate probable depression on the PHQ‐9 (≥10) reported a diagnosis of a depressive disorder, and 65% of those who scored above a cut off on the GAD‐7 taken to indicate GAD (≥10) reported having been given the diagnosis (≥10). The issue of traumatic stress symptoms being overlooked by mental health services has been highlighted as clinically problematic for decades, yet detection rates remain far from adequate. It has been reported that clinicians are reluctant to discuss traumatic events for fear of exacerbating symptoms or because of uncertainty regarding the management of PTSD with evidence that PTSD diagnosis in psychiatric settings is positively related to clinician confidence in the ability to effectively treat traumatic stress symptoms.^60^, ^61^ Increasing clinician confidence in their ability to ask about trauma history and offer appropriate evidence‐based treatment is necessary to improve patient outcomes. Although there are effective treatments for PTSD and comprehensive guidelines for its management,^62^, ^63^ most studies that informed the evidence base excluded participants with comorbidities such as severe depression, psychosis, and substance abuse.^58^ CPTSD has a different symptom profile to PTSD and there is little evidence that therapies currently recommended for PTSD effectively treat the DSO symptoms of CPTSD. Some specialist traumatic stress services offer phased‐based treatments where trauma focused therapy is preceded by stabilisation aimed at reducing self‐regulatory difficulties and followed by reintegration that supports re‐engagement in relationships, work, and social life. However, there is insufficient evidence to confidently recommend the best way to treat CPTSD and this work indicates an urgent need to develop, evaluate and disseminate appropriate interventions.^64^, ^65^, ^66^

## AUTHOR CONTRIBUTIONS

*Conception or design of the work*: Catrin Lewis, Katie Lewis, Alice Roberts, Bethan Edwards, Claudia Evison, Ann John, Alan Meudell, Holly Pearce, Natalie Richards, Ian Jones, Jonathan I. Bisson. *Acquisition of data*: Catrin Lewis, Katie Lewis, Alice Roberts, Holly Pearce, Natalie Richards, Ian Jones, Jonathan I. Bisson. *Data analysis*: Catrin Lewis. *Interpretation of data*: Catrin Lewis, Katie Lewis, Alice Roberts, Bethan Edwards, Claudia Evison, Ann John, Alan Meudell, Holly Pearce, Natalie Richards, Ian Jones, Jonathan I. Bisson. *Secured funding for the study*: Ann John, Ian Jones, Jonathan I. Bisson. Catrin Lewis led the drafting of the manuscript, but all authors contributed to the drafting and approved the final manuscript for submission.

## CONFLICT OF INTEREST

The authors declare no conflict of interest.

### PEER REVIEW

The peer review history for this article is available at https://publons.com/publon/10.1111/acps.13467.

## Supporting information


**APPENDIX S1**. Supporting Information

## Data Availability

Data for the current study is held by the National Centre for Mental Health (NCMH). NCMH welcome proposals for collaboration.
