# Supplementary material for: Trauma exposure and co‐occurring ICD‐11 post‐traumatic stress disorder and complex post‐traumatic stress disorder in adults with lived experience of psychiatric disorder
Source: Acta Psychiatr Scand. 2022 Jul 13;146(3):258–71. doi: 10.1111/acps.13467 (PMC9543812; doi:10.1111/acps.13467)
Supplement: Supplementary file 1 — APPENDIX S1. Supporting Information [file ACPS-146-258-s001.docx]

**Supplementary material**

**Table S1: Predictors of non-response to COVID-19 trauma survey - results of regression analyses (univariate)**

| Variable | OR | | 95% CI | | P |
| --- | --- | --- | --- | --- | --- |
| Age | 0.98 |  | | 0.98-0.99 | <.001 |
| Gender | 0.86 |  | | 0.75-0.99 | 0.035 |
| Ever employed | 0.67 |  | | 0.58-0.77 | <.001 |
| Minority ethnicity | 1.59 |  | | 1.16-2.17 | 0.004 |
| Diagnosis of bipolar disorder | 1.29 |  | | 1.08-1.54 | 0.004 |
| Diagnosis of schizophrenia | 1.35 |  | | 1.06-1.70 | 0.014 |
| Diagnosis of PTSD/CPTSD | 1.02 |  | | 0.85-1.21 | 0.852 |

Age – continuous; gender coded as 0 = male, 1 = female; ever employed coded as 1 = yes, 0 = no; minority ethnicity coded as 1 = yes, 0 = no; diagnosis of bipolar disorder coded as 1 = yes, 0 = no; diagnosis of schizophrenia coded as 1 = yes, 0 = no; diagnosis of PTSD/CPTSD coded as 1 = yes, 0 = no.

**Mental health diagnoses that could be endorsed by participants in the baseline survey**

*Mood Disorders*

Depressive disorder (Depression, Major Depression)

Bipolar disorder (Manic depression)

Mania/Hypomania

Premenstrual dysphoric disorder (PMDD) / Premenstrual syndrome (PMS)

*Psychotic Disorders*

Schizophrenia

Schizoaffective disorder

Psychosis

*Anxiety Disorders*

Anxiety (Generalised Anxiety Disorder, GAD)

Agoraphobia

Panic disorder

Phobias

Obsessive Compulsive Disorder (OCD)

*Autistic Spectrum Disorders*

Autism

Asperger’s syndrome

*Eating Disorders*

Anorexia

Bulimia

Binge Eating Disorder

*Attention Deficit Hyperactivity Disorder (ADHD)*

*Post-Traumatic Stress Disorder (PTSD)*

*Complex Post-Traumatic Stress disorder (CPTSD)*

*Personality Disorders*

Borderline personality disorder (emotionally unstable personality disorder)

Other Personality Disorder

*Alcohol and drug problems*

Alcohol dependence / misuse

Dependence / misuse of other drugs

*Dementia*

*Perinatal/Postpartum disorders*

Mood disorder in pregnancy

Postpartum psychosis (Postnatal psychosis / Puerperal Psychosis)

Postnatal depression (Postpartum Depression)
